# Distinct Sympathoneural and Cardiovagal Domains of Arterial Baroreflex Function in Autonomic Synucleinopathies

**DOI:** 10.21203/rs.3.rs-9248308/v1

**Published:** 2026-05-11

**Authors:** David S. Goldstein

**Affiliations:** National Institute of Neurological Disorders and Stroke Intramural Research Program

**Keywords:** autonomic failure, baroreflex, Parkinson disease, pure autonomic failure, multiple system atrophy

## Abstract

**Background and Objectives:**

The arterial baroreflex has sympathoneural and cardiovagal efferent limbs. Whether quantitative measures represent interchangeable indicators of a single physiological construct has been unclear. Moreover, the extent to which results agree in the synucleinopathies pure autonomic failure (PAF), Parkinson disease with or without orthostatic hypotension (PD + OH, PD No OH), and multiple system atrophy (MSA) remains incompletely understood. In a retrospective observational study we comprehensively assessed physiological and neurochemical biomarkers of arterial baroreflex function in these disorders.

**Methods:**

Data for 10 baroreflex-sympathoneural and 6 baroreflex-cardiovagal indices were compared among 38 patients with PAF, 63 with PD + OH, 63 with PD No OH, and 72 with MSA, along with 44 controls with complete datasets.

**Results:**

Indices of baroreflex-sympathoneural and baroreflex-cardiovagal function were only weakly correlated. Within the two domains, correlations across subjects were much stronger. Among sympathoneural measures, pressure recovery time after the Valsalva maneuver, the logarithm of the total baroreflex area, and the fractional orthostatic increment in plasma norepinephrine best distinguished patients with autonomic synucleinopathies from controls. Among cardiovagal measures, the Baroslope derived from Phase II of the Valsalva maneuver and the logarithms of low- and high-frequency heart rate variability during supine rest were the most informative. No baroreflex measure separated PAF from PD + OH or MSA.

**Conclusions:**

Indices derived from blood pressure and interbeat interval responses during the Valsalva maneuver capture much of the physiologically meaningful variation in arterial baroreflex function and provide efficient assessment of the sympathoneural and cardiovagal limbs in autonomic synucleinopathies.

## Brief Summary

Arterial baroreflex indices cluster into sympathoneural and cardiovagal domains. PRT, baroreflex areas, and Baroslope best capture dysfunction, which is especially prominent in PAF and PD + OH.

## Introduction

Cardiovascular autonomic failure is a defining and disabling feature of several neurodegenerative diseases linked to pathological accumulation of alpha-synuclein. Collectively these disorders have been referred to as autonomic synucleinopathies [21]. Prominent examples are Parkinson disease with orthostatic hypotension (PD + OH), pure autonomic failure (PAF) [1, 8, 16], and multiple system atrophy (MSA). Orthostatic hypotension (OH) in these conditions is associated with substantial morbidity, including falls, syncope, cognitive impairment, and with reduced survival [5].

The arterial baroreflex is the principal neural feedback mechanism for short-term regulation of arterial blood pressure. Through coordinated autonomic adjustments of heart rate, cardiac contractility, and vascular tone, the baroreflex keeps arterial pressure within bounds in the face of perturbations such as standing, exercise, and the Valsalva maneuver [11]. Impairment of baroreflex function is a defining feature of many disorders of autonomic regulation, including synucleinopathies, and contributes directly to orthostatic intolerance, syncope, and blood pressure lability.

Baroreflex function is not unitary. Rather, it comprises a sympathoneural component that regulates resistance vessels and thereby modulates vascular tone and blood pressure and a cardiovagal component that modulates heart rate responses to changes in arterial pressure [12, 17]. These components can be assessed quantitatively using physiological responses to the Valsalva maneuver, orthostatic challenge, and analyses of heart rate variability during supine rest [6, 9, 15]. Both limbs are often impaired in synucleinopathies. Lewy body diseases (LBDs) feature predominant sympathetic noradrenergic denervation or dysfunction—particularly in the heart [13, 22]. In MSA, impairment originates centrally, involving the caudal brainstem and intermediolateral cell column of the spinal cord, with relative sparing of sympathetic cardiac innervation in most patients [2, 4, 26, 30].

Although many quantitative indices of baroreflex function are available, their relationships to one another are not well established. This issue has practical importance for both research and clinical evaluation, because studies often report values for only a subset of available indices, and it is often assumed that different indices are interchangeable measures of the same physiological construct.

We report here the results of a retrospective observational cohort study of participants with synucleinopathies who had been evaluated at the National Institutes of Health (NIH) Clinical Center.

This study provides a systematic comparison of sixteen physiological and neurochemical baroreflex indices in a large cohort of autonomic synucleinopathies, enabling evaluation of the internal structure and relative informativeness of baroreflex measures.

We analyzed these indices of arterial baroreflex function in subjects representing six diagnostic groups: Control subjects, PAF, PD + OH, Parkinson disease without orthostatic hypotension (PD No OH), the cerebellar form of MSA (MSA-C), and the parkinsonian form of MSA (MSA-P).

The indices included ten measures reflecting baroreflex-sympathoneural function and six measures reflecting baroreflex-cardiovagal function. By addressing the following questions, the study aimed to identify the most physiologically informative baroreflex indices and to clarify how different autonomic disorders affect the sympathoneural and cardiovagal components of the arterial baroreflex.

1. Do indices of baroreflex-sympathoneural function agree across subjects?
2. Do indices of baroreflex-cardiovagal function agree across subjects?
3. Do sympathoneural baroreflex measures agree with cardiovagal measures?
4. Which diagnostic groups differ and in what ways in terms of baroreflex-sympathoneural or baroreflex-cardiovagal function?
5. Overall, which indices provide the most informative measures of baroreflex function?

## Methods

### Participants

All the participants provided written informed consent before accrual into IRB-approved protocols. Healthy volunteers had no prior history of neurological or autonomic disease, no vasoactive medications, and normal resting cardiovascular examinations. Disease groups were classified using established consensus criteria: Parkinson disease using the UK Brain Bank criteria [20], MSA using the second consensus criteria [7], and PAF defined by neurogenic orthostatic hypotension with evidence of peripheral noradrenergic deficiency and no evidence of a central neurodegenerative movement disorder or cognitive impairment.

Subjects were categorized according to the diagnostic group recorded in the dataset (Supplementary Data). Three control designations in the spreadsheet (Control healthy volunteers, Control PDRisk study participants who failed to meet criteria for further study, and Control participants referred for but without evidence of chronic autonomic failure (r/o CAF) were combined into a single Control group. All Control subjects lacked clinical evidence of autonomic failure, had no neurogenic orthostatic hypotension, and showed normal results on standard autonomic function testing. The final analysis therefore included six groups: Control, PAF, PD + OH, PD No OH, MSA-C, and MSA-P.

### Baroreflex Assessments

Beat-to-beat blood pressure was recorded using an automated finger cuff system Finapres Nova (Finapres Medical Systems BV, Amsterdam, The Netherlands) or Nexfin (bmeye BV, Amsterdam, The Netherlands; later Edwards Lifesciences, Irvine, CA, USA) during standardized autonomic testing [11].

Forearm blood flow was measured as done previously by our group [14, 19] via venous occlusion plethysmography using a mercury-in-silastic strain-gauge and a compressor for rapid brachial cuff inflation (D. E. Hokanson, Inc., Issaquah, WA). The strain gauge was placed around the upper forearm and connected to the plethysmograph. Subjects rested supine with the arm at about heart level. For each blood flow measurement the brachial cuff was inflated to above venous but below diastolic pressure (40 mmHg) to occlude venous outflow while allowing arterial inflow to continue. A wrist cuff was not used. Forearm blood flow expressed as milliliters per 100 mL of forearm tissue per minute (mL·100 mL^−1^·min^−1^) was calculated from the linear portion of the volume increase after the transient immediately following cuff inflation. At each sampling point forearm blood flow was measured six times, and the mean of the six determinations was used for analysis.

Baroreflex area during the depressor (Phase II) and recovery (Phase IV) limbs of the Valsalva maneuver was computed as the area under the systolic blood pressure curve below baseline [27]. Pressure recovery time (PRT) was measured from the pressure nadir after release of the maneuver to the return of blood pressure to baseline [11]. Neurochemical sympathoneural indices included the fractional orthostatic increments in plasma norepinephrine (Fx ΔNE) and 3,4-dihydroxyphenylglycol (FxΔDHPG) with head-up tilt for up to 5’ [10].

Baroreflex sensitivity (BRS-A) was calculated from the ratio of the blood pressure recovery time to the preceding decrease in pressure evoked by the Valsalva maneuver [28].

Sixteen quantitative indices of arterial baroreflex function were analyzed. Ten indices represented baroreflex-sympathoneural function: PRT [29], BRS-A [28], Log Baro. Area II [27], Log Baro. Area III, Log Baro. Area Total, ΔBPs Val Phase II, the fractional increment in forearm vascular resistance during orthostasis (FxΔFVR Ortho), the fractional increment in calculated total peripheral resistance during orthostasis (FxΔTPR Ortho based on application of the Beatscope algorithm [24], FxΔNE Ortho [10], and FxΔDHPG Ortho.

Six indices represented baroreflex-cardiovagal function: the increase in heart rate during Phase II of the Valsalva maneuver (Inc. in HR Val), the Baroslope (the slope of the line of best fit for the relationship between interbeat interval and systolic pressure during Phase II [15], the standard deviation of the electrocardiographic RR interval during supine rest (SDRR), log BL LF [9], log BL HF, and ΔHR/ΔBPs Ortho after up to 5’ of head-up tilt [25].

### Statistical Analysis

Agreement among indices across subjects was assessed after orienting all indices so that larger values consistently represented greater physiological abnormality. Indices for which lower values indicated greater abnormality, such as Baroslope, SDRR, log BL LF, log BL HF, and Inc. in HR Val, were multiplied by - 1 before correlation analysis. Because Shapiro-Wilk testing showed that 15 of the 16 indices deviated significantly from normality, correlation analyses among baroreflex indices were based primarily on Spearman rank-order correlation coefficients (ρ). Pearson correlation coefficients were also calculated as a secondary, sensitivity analysis to evaluate whether the overall correlation structure depended materially on the choice of correlation metric.

Separate correlation analyses were performed for baroreflex-sympathoneural indices, baroreflex- cardiovagal indices, and the combined set of indices spanning both domains. To examine concordance at the level of disease groups, correlations among indices were also calculated using group medians across the six diagnostic groups. Correlation matrices were displayed for Spearman analyses.

The structure of relationships among indices was visualized using force-directed network (FDN) representations derived from the correlation matrices. In the primary network analyses, edges were based on Spearman correlations; corresponding Pearson-based networks were generated for comparison. Each index was represented as a node, and each pairwise association was represented as an edge weighted by the absolute value of the correlation coefficient, such that more strongly associated indices were positioned closer together. The thickness of connecting lines was scaled to the magnitude of the correlation coefficient, and dashed edges indicated negative correlations. The network analyses were descriptive and intended to visualize the internal structure of relationships among indices rather than to establish causal relationships.

Parametric tests were applied when distributions approximated normality, whereas nonparametric methods were used for variables that deviated substantially from normal distributions. Group differences were assessed using Kruskal-Wallis tests followed by pairwise Mann-Whitney comparisons with Holm correction for multiple comparisons, or using analysis of variance with Tukey’s post-hoc tests, as appropriate. The pooled Control group served as the reference group for determining abnormality of indices in the disease groups.

A p value less than 0.05 defined statistical significance.

## Results

### Participants and Demographics

In the pooled dataset, the six analytic groups comprised 130 Control subjects, 63 subjects with PD No OH, 63 with PD + OH, 38 with PAF, 53 with MSA-P, and 19 with MSA-C (Supplementary File). Complete baroreflex datasets were available in 44 Control subjects. Compared with the pooled Control group, subjects with PD No OH, PD + OH, PAF, and MSA-P were older, whereas subjects with MSA-C did not differ significantly in age. Median age was 56 years in the Control group, 64 years in PD No OH, 70 years in PD + OH, 70 years in PAF, 61 years in MSA-P, and 56 years in MSA-C. Sex distribution also differed across groups. The Control group was 45% male, whereas the proportions of male subjects were higher in PD No OH (71%), PD + OH (71%), and PAF (68%). After Holm adjustment, the excess male predominance remained significant in PD No OH, PD + OH, and PAF, but not in MSA-P or MSA-C. Thus, the disease groups with the strongest autonomic phenotypes, especially PD + OH and PAF, tended to be older and more male-predominant than the pooled Control group.

### Normality of distributions

Shapiro-Wilk testing confirmed that 15 of the 16 indices deviated significantly from normality (all p < 0.05). The sole exception was log BL HF (W = 0.99, p = 0.096). This finding retrospectively validates the predominant use of non-parametric methods for group comparisons and supports the use of Spearman rank-order correlations as the primary approach for analyses of agreement among baroreflex indices. Pearson correlations were examined secondarily and yielded a similar overall structure of relationships (Supplementary Data file).

### Group comparisons in baroreflex indices

**Figure 1:**
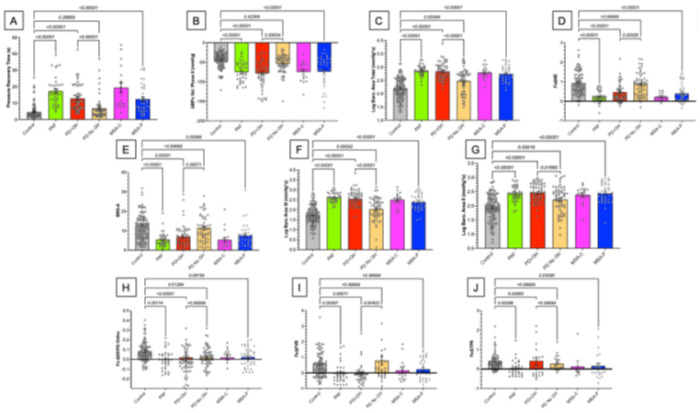
Baroreflex-sympathoneural indices across synucleinopathy groups. Individual values are shown for ten indices of baroreflex-sympathoneural function in Control subjects (gray) and in patients with the synucleinopathies pure autonomic failure (PAF, green), Parkinson disease with orthostatic hypotension (PD+OH, red), Parkinson disease without orthostatic hypotension (PD No OH, tan), cerebellar multiple system atrophy (MSA-C, magenta), and parkinsonian multiple system atrophy (MSA-P, blue). Panels show the following indices: (A) pressure recovery time (PRT), (B) ΔBPs during Valsalva Phase II, (C) log baroreflex area total, (D) fractional increase in plasma norepinephrine during orthostasis (FxΔNE Ortho), (E) BRS-A, (F) log baroreflex area III, (G) log baroreflex area II, (H) fractional increase in plasma DHPG during orthostasis (FxΔDHPG Ortho), (I) fractional change in forearm vascular resistance during orthostasis (FxΔFVR Ortho), and (J) fractional change in total peripheral resistance during orthostasis (FxΔTPR Ortho). Bars indicate mean values ± SEM. Horizontal brackets show comparisons between groups; numbers in italics show *p* values from Tukey’s post-hoc tests. Overall, the indices show marked abnormalities in PAF and PD+OH, with more limited or variable abnormalities in PD No OH and MSA, illustrating differing patterns of baroreflex-sympathoneural dysfunction across synucleinopathy phenotypes.

**Figure 2:**
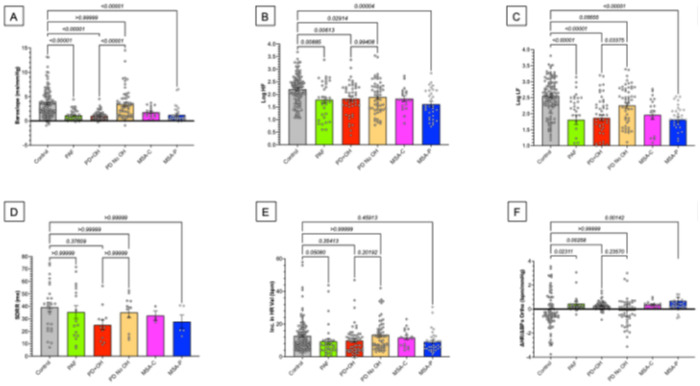
Baroreflex-cardiovagal indices across synucleinopathy groups. Individual values are shown for six indices of baroreflex-cardiovagal function in Control subjects (gray) and in patients with the synucleinopathies pure autonomic failure (PAF, green), Parkinson disease with orthostatic hypotension (PD+OH, red), Parkinson disease without orthostatic hypotension (PD No OH, tan), cerebellar multiple system atrophy (MSA-C, magenta), and parkinsonian multiple system atrophy (MSA-P, blue). Panels show the following indices: (A) increase in heart rate during the Valsalva maneuver (Inc. in HR Val), (B) baroreflex slope (Baroslope), (C) standard deviation of R–R intervals (SDRR), (D) log baseline low-frequency heart rate variability (log BL LF), (E) log baseline high-frequency heart rate variability (log BL HF), and (F) ΔHR/ΔBPs during orthostasis. Bars indicate mean values ± SEM. Horizontal brackets show comparisons between groups; numbers in italics show *p* values from Tukey’s post-hoc tests. Overall, the indices show reduced cardiovagal baroreflex function in the synucleinopathies, with the most consistent abnormalities in PAF and PD+OH and more variable changes in PD No OH and MSA.

Significant overall group effects were observed for 15 of the 16 indices. SDRR was the only measure that did not differ significantly across groups. Relative to the Control group, PAF and PD + OH each showed abnormalities in 13 or more indices, indicating severe impairment of baroreflex function (Figs. 1 and 2). MSA-P showed abnormalities in 13 indices and MSA-C in 12 indices. PD No OH exhibited a milder pattern, with abnormalities in 6 indices (Figs. 1 and 2).

In PAF, 15 of the 16 indices differed significantly from Control subjects. The only index that did not differ significantly was SDRR. In PD + OH, 13 indices were abnormal relative to Control subjects, whereas FxΔTPR Ortho, Inc. in HR Val, and SDRR did not differ significantly.

PD No OH showed abnormalities in 6 indices, including PRT, the three baroreflex area measures, FxΔDHPG Ortho, and log BL HF, while the remaining indices were not significantly different from Control subjects.

In MSA-C, 12 indices differed significantly from Control subjects. FxΔFVR Ortho, FxΔDHPG Ortho, Inc. in HR Val, and SDRR did not differ significantly.

In MSA-P, 13 indices were abnormal relative to Control subjects, whereas FxΔFVR Ortho, Inc. in HR Val, and SDRR did not differ significantly.

Direct comparison between PD + OH and PD No OH revealed significant differences in 10 indices. PD + OH showed more severe abnormalities in PRT, BRS-A, the three baroreflex area measures, ΔBPs Val Phase II, FxΔFVR Ortho, FxΔNE Ortho, Baroslope, and log BL LF (Figs. 1 and 2). The two PD groups did not differ significantly in FxΔTPR Ortho, FxΔDHPG Ortho, Inc. in HR Val, SDRR, log BL HF, or ΔHR/ΔBPs Ortho.

Controls with complete data across all 16 indices (n = 23) were significantly older than controls with incomplete datasets (n = 107; median age 60.4 vs. 54.5 years, Mann-Whitney p = 0.020). Sex distribution did not differ significantly (40% vs. 47% male, χ² p = 0.66). The age difference reflects the greater likelihood of older control subjects having undergone the full testing battery, including forearm blood flow and neurochemical sampling.

To assess whether the observed group differences reflected demographic confounding, Kruskal-Wallis tests were repeated on residuals after regressing out age and sex. All 15 indices that showed significant overall group effects in the unadjusted analyses remained significant after adjustment. SDRR remained non-significant both before and after adjustment. The significance status of no index changed, indicating that the group differences in baroreflex function are robust to demographic adjustment. Partial Spearman correlations with disease status (disease vs. control), after controlling for age and sex, remained significant for 13 of 16 indices. Three indices — Inc. in HR Val, SDRR, and ΔHR/ΔBPs Ortho — lost significance after adjustment, suggesting their apparent associations with disease were partly attributable to demographic differences rather than baroreflex dysfunction per se.

ROC analyses were performed for each index separately, comparing each disease group against the pooled Control group; these analyses addressed discrimination between each disease group and controls, not among disease groups themselves. ROC analysis confirmed that no single index reliably distinguished among the three major autonomic synucleinopathy groups after Holm correction for multiple comparisons, supporting the conclusion that baroreflex assessments alone are insufficient for individual differential diagnosis. Nonetheless, several indices showed high discriminative accuracy for distinguishing patients with autonomic synucleinopathies from controls. PRT (AUC = 0.946), Log Baro. Area III (AUC = 0.956), and Log Baro. Area Total (AUC = 0.944) were particularly informative in PAF; PRT (AUC = 0.899) and Log Baro. Area III (AUC = 0.914) performed similarly well in PD + OH. Discriminative accuracy was substantially lower in PD No OH, with no index exceeding an AUC of 0.68, consistent with the milder baroreflex impairment in that group. Among cardiovagal indices, Baroslope (AUC = 0.818 for PD + OH) and log BL LF (AUC = 0.840 for MSA-P) showed the strongest discriminative performance. When pairwise comparisons among disease groups were examined, no index significantly distinguished PAF from MSA-P, PAF from MSA-C, PD + OH from MSA-P, or PD + OH from PAF after Holm correction, formally confirming that baroreflex indices cannot reliably differentiate among these disorders at the individual level.

### Agreement among Baroreflex Measures

Across subjects, directionality-normalized baroreflex-sympathoneural indices showed substantial internal agreement by Spearman analysis, indicating concordance of abnormality across several physiological and neurochemical biomarkers of sympathetic baroreflex function. The strongest relationships were observed among Log Baro. Area II, Log Baro. Area III, and Log Baro. Area Total, which formed a tightly intercorrelated cluster. PRT was also strongly associated with these variables. By contrast, the orthostatic vascular resistance measures FxΔFVR Ortho and FxΔTPR Ortho were only weakly correlated with most of the other sympathoneural indices. Thus, agreement among sympathoneural measures was driven primarily by a core cluster consisting of PRT and the baroreflex area indices.

The baroreflex-cardiovagal indices likewise showed internal coherence by Spearman analysis, with the strongest associations among log BL LF, log BL HF, SDRR, and Baroslope. Inc. in HR Val showed moderate correlations with this group, whereas ΔHR/ΔBPs Ortho showed weaker or, in some comparisons, inverse relationships with the other cardiovagal indices.

Relationships between sympathoneural and cardiovagal indices were weaker than relationships within each domain. Across the combined matrix, most cross-domain relationships were modest in magnitude, with median absolute correlation coefficients of approximately 0.17 by Spearman and 0.11 by Pearson, although a few pairwise associations reached the moderate range (up to about 0.5).

When correlations were calculated using group medians across the six diagnostic groups, concordance among indices increased markedly, indicating that disease-group effects accentuate the shared signal within each physiological domain.

Pearson correlation analyses yielded a broadly similar pattern, with preservation of the same main clusters and the same distinction between stronger within-domain and weaker cross-domain relationships. Because of the markedly skewed distributions of most indices, however, the Spearman results were considered primary and the Pearson analyses supportive.

### Network visualizations

**Figure 5:**
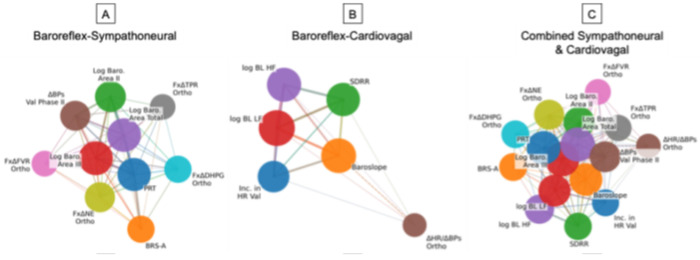
Network representations of relationships among indices of arterial baroreflex-sympathoneural and baroreflex-cardiovagal function. The panels show force-directed networks (FDNs). Each circle represents one index, and the spatial arrangement of circles reflects the strength of correlations among indices across all subjects. Circle size is proportional to the summed absolute correlations of that index with the other indices, with larger circles representing variables that are more strongly associated overall with the rest of the network. Lines connecting circles represent correlations between indices; line thickness is proportional to the magnitude of the correlation coefficient, with thicker lines indicating stronger associations and thinner lines indicating weaker associations; solid lines indicate positive correlations and dashed lines indicate negative correlations. (A) Spearman FDN for baroreflex-sympathoneural indices; (B) Spearman FDN for baroreflex-cardiovagal indices; (C) Spearman FDN for combined sympathoneural and cardiovagal indices. Abbreviations: PRT, pressure recovery time during the Valsalva maneuver; ΔBPs Val Phase II, change in systolic blood pressure during Phase II of the Valsalva maneuver; BRS-A, baroreflex sensitivity estimated from the ratio of the pressure recovery time to the decrease in systolic blood pressure during the Valsalva maneuver; Log Baro. Area II, Log Baro. Area III, and Log Baro. Area Total, logarithm of the baroreflex area relating R–R interval to systolic blood pressure during phases II and III of the Valsalva maneuver and across the entire maneuver; FxΔFVR Ortho, fractional change in forearm vascular resistance during orthostasis; FxΔTPR Ortho, fractional change in total peripheral resistance during orthostasis; FxΔNE Ortho, fractional increase in plasma norepinephrine during orthostasis; FxΔDHPG Ortho, fractional increase in plasma dihydroxyphenylglycol during orthostasis; Inc. in HR Val, increase in heart rate during the Valsalva maneuver; Baroslope, baroreflex slope relating heart rate or R–R interval to systolic blood pressure; SDRR, standard deviation of R–R intervals; log BL LF, logarithm of baseline low-frequency heart rate variability; log BL HF, logarithm of baseline high-frequency heart rate variability; ΔHR/ΔBPs Ortho, ratio of the change in heart rate to the change in systolic blood pressure during orthostasis.

Network analysis revealed that when the FDNs were constructed separately for the baroreflex- sympathoneural and baroreflex-cardiovagal limbs, the internal consistencies within the two components were clear. In the sympathoneural network (Fig. 5A), the most strongly connected cluster consisted of PRT and the baroreflex area measures, with additional links to ΔBPs Val Phase II. The neurochemical indices FxΔNE Ortho and FxΔDHPG Ortho were moderately connected to this cluster, whereas the vascular resistance measures FxΔFVR Ortho and FxΔTPR Ortho were positioned more peripherally with fewer and weaker connections. BRS-A also showed relatively limited connectivity compared with the baroreflex area and PRT measures.

The baroreflex-cardiovagal network showed a more compact structure centered on Baroslope and indices of heart rate variability (Fig. 5B). Log BL LF, Log BL HF, and SDRR formed a tightly interconnected cluster with strong mutual correlations and strong connections to Baroslope. Inc. in HR Val showed moderate connectivity to this cluster, while ΔHR/ΔBPs Ortho was positioned more peripherally, with fewer and weaker connections to the other cardiovagal indices.

Network analysis of the sympathoneural and cardiovagal indices combined revealed that the most highly connected variables across the two baroreflex efferent limbs were Log Baro. Area Total, Log Baro. Area III, Baroslope, log BL LF, Log Baro. Area II, and PRT (Fig. 5C). Several indices were only weakly connected to other measures. FxΔFVR Ortho, FxΔTPR Ortho, and ΔHR/ΔBPs Ortho showed relatively few and weak correlations with the other indices.

## Discussion

The present analysis demonstrates that indices of arterial baroreflex function segregate into two partially independent physiological domains corresponding to the sympathoneural and cardiovagal efferent limbs. This helps explain why studies using different subsets of baroreflex indices may reach only partly overlapping conclusions. Within the sympathoneural domain, the strongest relationships were observed among PRT and the baroreflex area-based measures associated with the Valsalva maneuver. Orthostatic vascular resistance indices showed weaker associations with other sympathoneural measures. Within the cardiovagal domain, the strongest coherence involved LF and HF power of heart rate variability in the frequency domain, SDRR in the time domain, and Baroslope. Since cross-domain correlations were only modest, the findings indicate that the sympathoneural and cardiovagal components of the arterial baroreflex are related but not interchangeable physiological processes.

Taken together, the findings indicate that a relatively small subset of indices captures most of the physiologically meaningful information in the baroreflex dataset. Measures such as PRT, the baroreflex area indices, ΔBPs Val Phase II, Baroslope, and LF-related cardiovagal measures appear to provide the most informative summary of baroreflex function. These results support the use of a small panel of integrated baroreflex indices for physiological phenotyping of autonomic disorders.

Group comparisons revealed extensive abnormalities of baroreflex function in PAF, PD + OH, and both types of MSA, with milder abnormalities in PD No OH. The results reinforce the view that baroreflex assessments alone are insufficient for individual differential diagnosis among PAF, PD + OH, and MSA [18]. Other modalities such as positron emission tomography to visualize putamen and cardiac catecholaminergic innervation [23] or analyses of alpha-synuclein seeding [8] seem required.

Correlations among indices differed markedly when calculated across individual subjects as opposed to across diagnostic groups. Across subjects, relationships between sympathoneural and cardiovagal measures were rather weak; correlations based on group medians were substantially stronger, in the range of approximately 0.8 to 0.9. This pattern likely reflects substantial physiological heterogeneity among individuals, whereas the shared pathophysiological effects of autonomic synucleinopathies become clear when results are considered at the level of disease groups.

The baroreflex-sympathoneural cluster was not purely physiological. The moderate connectivity of FxΔNE Ortho with the core Valsalva-derived cluster suggests convergence of physiological and neurochemical biomarkers on a common baroreflex-sympathoneural abnormality.

### Limitations

Limitations include variable biomarker availability across subjects. In addition, this is a single-center referral cohort, which may introduce selection bias toward more severe phenotypes.

The cross-sectional design cannot establish the temporal sequence of sympathoneural versus cardiovagal failure; longitudinal studies are needed to determine whether sympathoneural impairment precedes cardiovagal impairment in autonomic synucleinopathies.

Several additional limitations pertain specifically to the baroreflex measures analyzed. First, some of the indices are mathematically or physiologically related. For example, the baroreflex area measures and PRT are derived from related features of the Valsalva maneuver blood pressure response, and some cardiovagal indices are derived from overlapping aspects of heart rate variability. Such relationships may partially inflate correlations among indices and therefore increase the apparent internal concordance within physiological domains.

Some measures were based primarily on responses during the Valsalva maneuver, whereas others reflected orthostatic responses or spectral properties of heart rate variability during supine rest. Differences in stimulus conditions and underlying physiological mechanisms could introduce variability unrelated to baroreflex integration itself and may contribute to weaker correlations between some indices.

Another limitation concerns the calculated orthostatic vascular resistance indices, especially FxΔTPR Ortho. Total peripheral resistance was derived from stroke volume estimated by the Beatscope algorithm from the finger arterial pressure waveform. In conditions with marked digital vasoconstriction, local changes in arterial stiffness and wave reflection [3] could distort waveform morphology and thereby affect the accuracy of noninvasively estimated blood pressure and stroke volume. Consistent with this concern, discrepancies have been reported between invasive and non-invasive blood pressure measurements at higher doses of infused norepinephrine, attributed in part to altered vascular tone, arterial stiffness, and wave reflection [31]. Therefore, some of the variability or weak correlations of FxΔTPR Ortho, and possibly of FxΔFVR Ortho, with other measures of baroreflex-sympathoneural function may reflect methodological issues in addition to physiological heterogeneity.

Since the correlation, network, and multi-dimensional scaling analyses were descriptive, they do not establish causal relationships among physiological variables. Measures that appear centrally connected in network representations should be interpreted as indices that co-vary with other measures rather than as necessarily representing the primary physiological drivers of arterial baroreflex function or dysfunction.

Disorders such as PD and MSA can vary substantially in disease duration, severity, and distribution of autonomic involvement. Such heterogeneity may attenuate correlations among baroreflex indices across subjects and complicate interpretation of group-level differences.

Controls with complete data across all 16 indices were significantly older than controls with incomplete datasets, whereas sex distribution did not differ significantly. The age discrepancy likely reflects greater likelihood of older control subjects having undergone the full testing battery. Age-related differences in autonomic function therefore could have influenced the observed correlation structure.

Finally, overall baroreflex function reflects input not only from high pressure arterial baroreceptors but also low pressure (“volume”) baroreceptors located in the pulmonary veins and atria. Routine clinical measures of cardiopulmonary (“low-pressure”) baroreflex function are not available.

## Conclusions and Implications

This study demonstrates that quantitative indices of arterial baroreflex function cluster into two partially independent physiological domains corresponding to the sympathoneural and cardiovagal limbs. Within the sympathoneural domain, the strongest agreement is observed among PRT and the baroreflex area measures derived from the Valsalva maneuver; within the cardiovagal domain the strongest relationships involve the Baroslope and spectral measures of heart rate variability. Cross-domain relationships were comparatively modest, indicating that the sympathoneural and cardiovagal components of the baroreflex are related but not interchangeable physiological processes. Across diagnostic groups, the indices showed strong concordance, with the most extensive abnormalities observed in PAF and PD + OH, intermediate abnormalities in MSA, and more limited abnormalities in PD No OH. Collectively, these findings suggest that a relatively small subset of indices captures much of the physiologically meaningful variation in arterial baroreflex function in synucleinopathies.

The present findings have practical implications for physiological research and clinical autonomic testing. Because several indices convey largely overlapping information, a reduced set of measures seems sufficient to characterize baroreflex function in most settings. Indices such as PRT, baroreflex areas, ΔBPs Val Phase II, Baroslope, and LF-related measures appear to provide the most informative representation of arterial baroreflex regulation. The separation between sympathoneural and cardiovagal domains also underscores the importance of assessing both components when evaluating autonomic disorders. The pattern of abnormalities across disease groups highlights that PAF and PD + OH involve similarly severe baroreflex impairment.

## Supplementary Material

This is a list of supplementary files associated with this preprint. Click to download.

• Baro.msSupplementaryDataforCAR.xlsx

• NIHPublishingAgreementCoverSheetCAR.docx

## Figures and Tables

**Figure 3:**
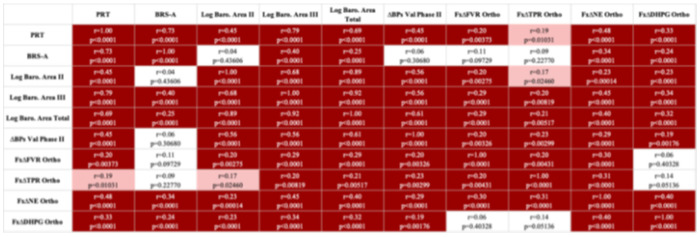
Correlation matrix heatmap for indices of arterial baroreflex-sympathoneural function. Spearman correlation coefficients and associated *p* values are shown for ten indices of baroreflex-sympathoneural function across all subjects. Each cell displays the correlation coefficient and the corresponding *p* value; darker red shading and white text indicate cells where the *p* value is less than 0.01. The indices include both physiological and neurochemical measures. PRT denotes pressure recovery time during the Valsalva maneuver and BRS-A baroreflex sensitivity estimated from the ratio of the pressure recovery time to the decrease in systolic blood pressure during the Valsalva maneuver (BRS-A method). Log Baro. Area II and Log Baro. Area III are the logarithm of the baroreflex area relating R–R interval to systolic blood pressure during Phases II and III of the Valsalva maneuverand Log Baro. Area Total the logarithm of the total baroreflex area across the phases of the maneuver. ΔBPs Val Phase II denotes the change in systolic blood pressure during Phase II of the maneuver; FxΔFVR Ortho the fractional change in forearm vascular resistance during orthostasis, and FxΔTPR Ortho the fractional change in total peripheral resistance during orthostasis. FxΔNE Ortho is the fractional increase in plasma norepinephrine and FxΔDHPG Ortho the fractional increase in plasma dihydroxyphenylglycol (DHPG) during orthostasis. Overall, the matrix shows good agreement among several but not all physiological indices of baroreflex-sympathoneural function.

**Figure 4:**
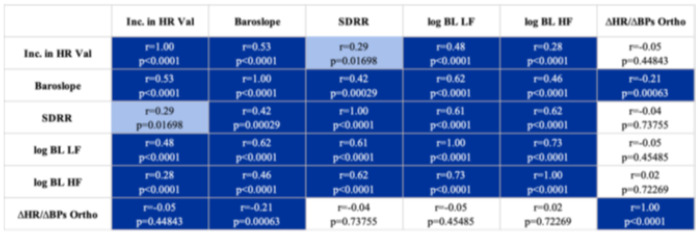
Correlation matrix heatmap for indices of baroreflex-cardiovagal function. Spearman correlation coefficients and associated p values are shown for six indices of baroreflex-cardiovagal function across all subjects. Each cell displays the correlation coefficient and the corresponding p value; dark blue shading and white text indicate cells where the p value is less than 0.01. Inc. in HR Val denotes the increase in heart rate during the Valsalva maneuver; Baroslope the baroreflex slope relating R–R interval to systolic blood pressure during the Valsalva maneuver; SDRR the standard deviation of R–R intervals during supine rest; log BL LF the logarithm of baseline low-frequency power of heart rate variability during supine rest; log BL HF the logarithm of baseline high-frequency power of heart rate variability during supine rest; and ΔHR/ΔBPs Ortho the ratio of the change in heart rate to the change in systolic blood pressure during head-up tilt at 90 degrees from horizontal. Overall, the matrix shows good agreement among several but not all cardiovagal indices, particularly between measures derived from heart rate variability and Baroslope.
